# Developing a flexible learning activity on biodiversity and spatial scale concepts using open‐access vegetation datasets from the National Ecological Observatory Network

**DOI:** 10.1002/ece3.7385

**Published:** 2021-03-21

**Authors:** Diane M. Styers, Jennifer L. Schafer, Mary Beth Kolozsvary, Kristen M. Brubaker, Sara E. Scanga, Laurel J. Anderson, Jessica J. Mitchell, David Barnett

**Affiliations:** ^1^ Western Carolina University Cullowhee NC USA; ^2^ Winthrop University Rock Hill SC USA; ^3^ Siena College Loudonville NY USA; ^4^ Hobart and William Smith Colleges Geneva NY USA; ^5^ Utica College Utica NY USA; ^6^ Ohio Wesleyan University Delaware OH USA; ^7^ Spatial Analysis Lab University of Montana Missoula MT USA; ^8^ National Ecological Observatory Network Battelle Memorial Institute Boulder CO USA

**Keywords:** big data, biodiversity metrics, quantitative skills, scaling, teaching, undergraduate education

## Abstract

Biodiversity is a complex, yet essential, concept for undergraduate students in ecology and other natural sciences to grasp. As beginner scientists, students must learn to recognize, describe, and interpret patterns of biodiversity across various spatial scales and understand their relationships with ecological processes and human influences. It is also increasingly important for undergraduate programs in ecology and related disciplines to provide students with experiences working with large ecological datasets to develop students’ data science skills and their ability to consider how ecological processes that operate at broader spatial scales (macroscale) affect local ecosystems. To support the goals of improving student understanding of macroscale ecology and biodiversity at multiple spatial scales, we formed an interdisciplinary team that included grant personnel, scientists, and faculty from ecology and spatial sciences to design a flexible learning activity to teach macroscale biodiversity concepts using large datasets from the National Ecological Observatory Network (NEON). We piloted this learning activity in six courses enrolling a total of 109 students, ranging from midlevel ecology and GIS/remote sensing courses, to upper‐level conservation biology. Using our classroom experiences and a pre/postassessment framework, we evaluated whether our learning activity resulted in increased student understanding of macroscale ecology and biodiversity concepts and increased familiarity with analysis techniques, software programs, and large spatio‐ecological datasets. Overall, results suggest that our learning activity improved student understanding of biological diversity, biodiversity metrics, and patterns of biodiversity across several spatial scales. Participating faculty reflected on what went well and what would benefit from changes, and we offer suggestions for implementation of the learning activity based on this feedback. This learning activity introduced students to macroscale ecology and built student skills in working with big data (i.e., large datasets) and performing basic quantitative analyses, skills that are essential for the next generation of ecologists.

## Graphical abstract

In this paper, we present the development, implementation, and outcomes of a classroom learning activity developed by an interdisciplinary team of ecologists and spatial scientists with the goal of teaching biodiversity and spatial scale concepts to undergraduate students. Overall, results suggest that our learning activity improved student understanding of biological diversity, biodiversity metrics, and patterns of biodiversity across several spatial scales. This learning activity introduced students to macroscale ecology and built student skills in working with big data and performing basic quantitative analyses, skills that are essential for the next generation of ecologists.

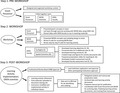

## 1 INTRODUCTION

It is essential that undergraduate students in ecology and other natural sciences develop a solid understanding of the concept of biological diversity. The Earth is undergoing a biodiversity crisis, with loss of species occurring at an unprecedented rate, largely from human impacts (IPBES, 2019; Ceballos et al., 2015), and understanding patterns and drivers of biodiversity is vital to developing potential solutions (Luque et al., 2018; Brooks et al., 2008; Medail & Quezel, 1999). Although biodiversity is a complex concept with multiple levels of organization, species diversity is the most typical way biodiversity is measured and assessed. However, species diversity can be difficult to comprehend because it can be measured in multiple ways (e.g., species richness, diversity indices), and several different metrics are used by scientists to interpret the distribution of biological diversity and how humans influence biodiversity patterns (Colwell, 2009; Hughes et al., 2008; Loreau, 2010; Petchey & Gaston, 2002; Tscharntke et al., 2012; Zimmermann et al., 2010).

Successfully teaching biodiversity metrics presents several challenges (Navarro‐Perez & Tidball, 2012). Species biodiversity is typically assessed at three spatial scales: local (alpha diversity), change in species composition across habitats within a region (beta diversity), and regional or landscape scale (gamma diversity, Angeler & Drakare, 2013; Loreau, 2010; Magurran, 2004; Tuomisto, 2010). Beta diversity is perhaps the most confusing of these three metrics because definitions of beta diversity vary (e.g., turnover in species, changes in species composition) and beta diversity metrics can appear disconnected from the definitions (Loreau, 2010; ShengBin et al., 2010). In fact, experts in the field debate methods for measuring beta diversity and their interpretation (Tuomisto, 2010). Furthermore, spatial scale is intrinsic to understanding beta diversity, and traditional biology programs often lack explicit instruction in spatial reasoning such as is gained from coursework in geography or geographic information systems (GIS) (Steinberg & Steinberg, 2015; Tilman & Kareiva, 2018).

Scale is fundamental to several disciplines, but defined in different ways, making it another challenging concept to teach (Cheek et al., 2017). Scale can be used to address space and/or time, or taught as a magnitude of a dimension or relationship between two objects or events. Because of the difficulties associated with teaching concepts related to scale, it may rarely be included as a topic in biology courses. In fact, Cheek et al. (2017) found only three studies that examined teaching and learning of scale in biology and ecology classrooms, indicating that more research is needed in this area.

It is increasingly important for undergraduate programs in ecology and related disciplines to teach students how to analyze large ecological datasets (Langen et al., 2014). Although there are challenges to incorporating big data into the undergraduate classroom (Langen et al., 2014), such as managing student frustration, there are many benefits. Skills and experience gained from participating in projects that use big data will help prepare a generation of ecologists to collaborate with colleagues from multiple disciplines (e.g., climate science, remote sensing) to solve global‐scale problems (Carey et al., 2019; Shiklomanov et al., 2019). Analysis of large datasets can help students understand how broad‐scale (macroscale) ecological processes affect local ecosystems (Carey et al., 2020; Heffernan et al., 2014), while gaining competence in big data management and analysis methods that are essential for future scientists in the field (Hampton et al., 2017). Using real, open‐access data collected at multiple spatial scales through observatory networks (e.g., National Ecological Observatory Network (NEON), Long‐term Ecological Research (LTER) sites, Critical Zone Observatories (CZOs)) can involve students in authentic science (Styers, 2018) as they engage with large datasets to understand biodiversity at multiple spatial scales.

To support the goals of improving student big data skills and their understanding of macroscale ecology and biodiversity at multiple spatial scales, we formed an interdisciplinary team to design a learning activity to teach macroscale concepts related to biodiversity using NEON data. Scientists and faculty from various fields (e.g., ecology, remote sensing, geography) who are associated with the Ecological Research as Education Network (EREN, erenweb.org) worked cooperatively to design and test a learning activity with sufficient flexibility to be incorporated into a variety of courses (e.g., ecology, conservation biology, GIS, remote sensing) across a range of student skill levels. We piloted this learning activity in six courses enrolling a total of 109 students, ranging from midlevel ecology and GIS/remote sensing courses to upper‐level conservation biology. While the majority of students were STEM majors, students had a diverse set of specialties within STEM and different background knowledge, ranging from environmental studies or natural resource management to biology. Our goal was to determine how to best use our learning activity to improve student understanding of macroscale ecology and biodiversity concepts, understanding of NEON’s large spatio‐ecological datasets, and skills in data management and use of software programs (e.g., spreadsheets, GIS, statistical software).

## 2 METHODS

### 2.1 Classroom learning activity development

**FIGURE 1:**
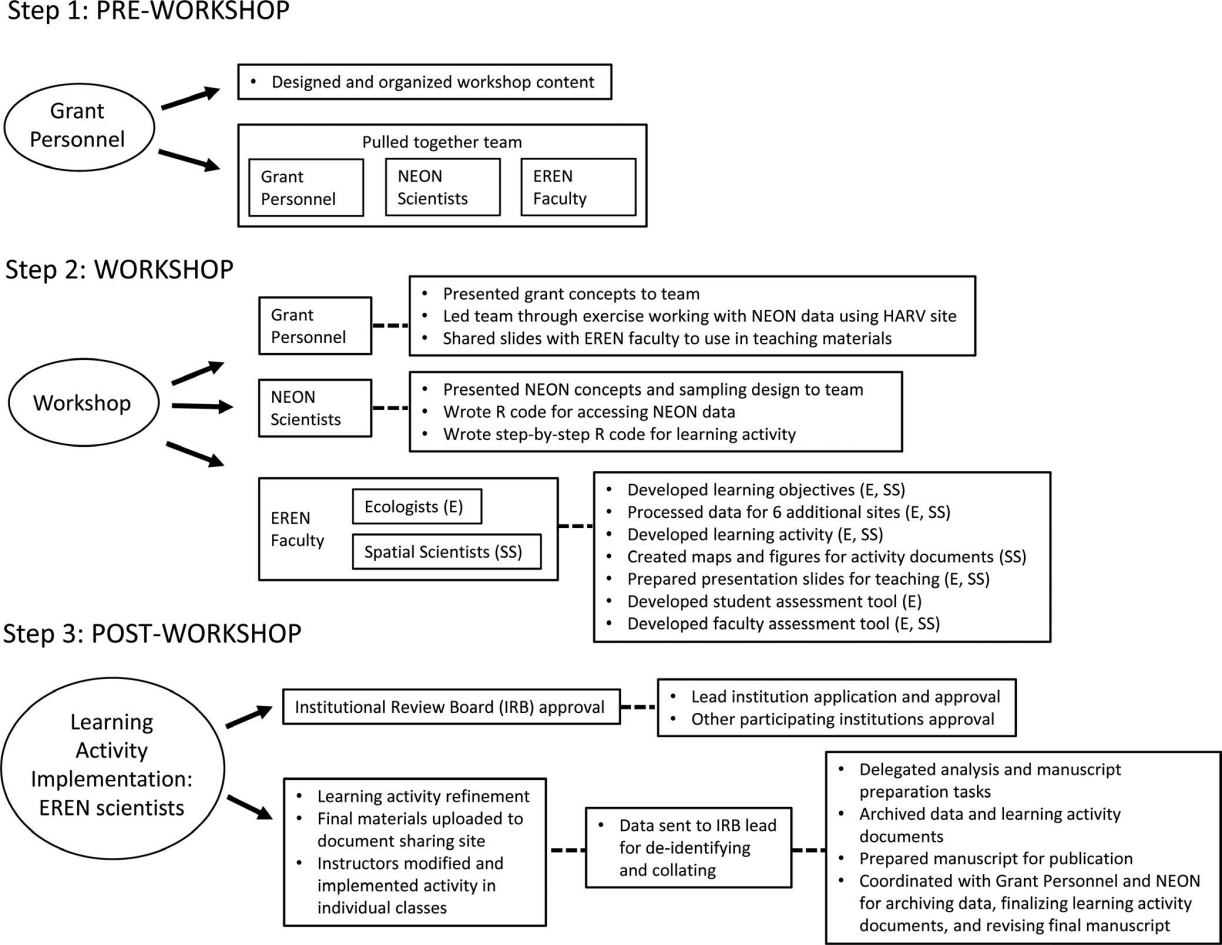
Conceptual diagram of the process for developing our classroom learning activity. Grant personnel designed and organized the workshop and pulled together the team of scientists. The workshop was conducted over two and a half days, in which a majority of the learning activity and associated materials were developed. The remaining work was completed over the following three weeks, and then, IRB review for the assessment of student learning was initiated. The learning activity was implemented in six different classrooms during the fall 2018 semester

Our team included grant personnel, NEON scientists, and EREN faculty from primarily undergraduate institutions (PUIs) specializing in both ecology and spatial sciences, all of which facilitated important knowledge sharing (Figure 1). This interdisciplinary team approach had several benefits, including providing support to faculty who may be less familiar with spatial tools and big data and adding important skills in spatial reasoning and ecological concepts to more traditional GIS classes (Bearman et al., 2016). The team approach brought some challenges as well—for example, the computing systems and computing support at our different colleges vary broadly, so we had to develop multiple versions of some of the course materials—but the benefits in making complex learning activities more accessible outweighed the drawbacks.

**FIGURE 2:**
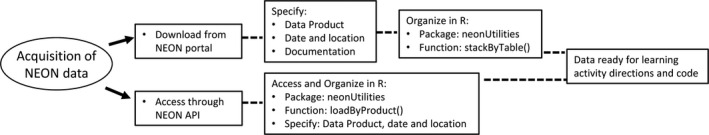
Conceptual diagram of the process for getting started with NEON data. Primary methods for accessing NEON organismal data include (1) download from the NEON Data Portal and (2) programmatic access from within the NEON application programming interface (API). Data via the manual download occurs at the NEON Data Portal (https://data.neonscience.org/data‐products/explore), requires a NEON data product name and/or number (e.g., “Plant presence and percent cover,” DP1.10058.001), date and location (state, NEON domain, or site), specification on inclusion of documentation such as protocol that guided data collection, and selection of the “basic” (primary measurements) or “expanded” package (related data and samples). These data download as a compressed folder with a nested by month and location folder structure. These are best organized programmatically with a NEON‐developed function (stackByTable()) in the neonUtilities package for the R programming language. Accessing the NEON data programmatically is accomplished through the NEON API also in R with the neonUtilities package. The function loadByProduct() requires the same data product, date range, location, documentation, and package specifications. The neonUtilities package is available via GitHub (https://github.com/NEONScience), a code hosting platform for version control and collaboration. Detailed instructions for the download of NEON data can be found in the NEON tutorials library (https://www.neonscience.org/resources/learning‐hub/tutorials/download‐explore‐neon‐data)

A workshop was designed and organized by Dr. Jessica Mitchell (University of Montana) and funded as part of an NSF‐sponsored research project, which the participants titled the Joint EREN‐NEON project (PI: Jessica Mitchell; NSF Grant No. 1916896). The workshop agenda and timeline, and classroom teaching and learning activity materials are available for download at https://drive.google.com/drive/folders/1CinmrXQ‐KCVqbtR6YLFNVd5_VgV8XAoY?usp=sharing. After introductions and overviews of EREN, NEON, biodiversity, and the NSF‐sponsored research project, participants were led through an exercise using plant presence and percent cover (PPPC) field data from the NEON Harvard Forest site (HARV) to calculate alpha and beta diversity. A NEON scientist familiar with the internal R scripts for downloading and organizing NEON data was available for questions and assistance (Lunch et al., 2020). The R code and workflow provided (see Figure 2) allowed participants to easily access the NEON data and prepare it for use in the dry run of the learning activity. The R code was tweaked on‐site based on real‐time feedback and is now available on NEON’s online data portal for anyone to use. The first day of the workshop ended with participants developing learning objectives for the classroom learning activity (Table 1).

**TABLE 1:** Learning objectives for the learning activity and minimum concepts covered by each faculty member that implemented the activity for the three topics covered in the activity

Topic	Learning objectives	Minimum concepts
Biodiversity metrics	1. Differentiate alpha, beta, and gamma diversity. 2. Recall the strengths and weaknesses of diversity metrics. 3. Calculate plant field diversity metrics (alpha, beta, and gamma diversity) for NEON plots/sites.	Alpha diversity Beta diversity Gamma diversity
Spatial scale	1. Describe the concept of multiple spatial scales in ecology. 2. Describe how ecological data collected at one scale can be “scaled up” or “scaled down” to describe ecological patterns. 3. Recognize the benefits of analyzing diversity metrics at multiple spatial scales. 4. Describe macroscale, differentiating it from other scales of inquiry. 5. Describe the nested plot sampling method for generating species–area curves. 6. Plot and interpret species–area curves.	Macrosystems Macroscale Scaling up and scaling down Value of analyzing multiple spatial scales when applying biodiversity metrics for conservation Nested plot sampling Species–area curves
NEON	1. Summarize how the objectives of the National Ecological Observatory Network (NEON) support macroscale science	Mission of NEON Application to macroscale science

On the second day of the workshop, participants completed biodiversity calculations for six additional NEON field sites, all located in the eastern deciduous forest biome. In total, there were two sites each from the Northeast (D01) and Mid‐Atlantic (D02) NEON domains and three sites from the Appalachians and Cumberland Plateau domain (D07). In addition to calculating alpha and beta diversity, participants calculated and compared gamma diversity for each of the seven sites. Faculty then used the afternoon to collectively develop the classroom learning activity, teaching materials, and assessment tools according to the agreed‐upon learning objectives. The final day of the workshop was used to perform a test run through the learning activity, develop a timeline for implementation, data sharing, and management, and discuss future collaborative opportunities.

Workshop participants developed a set of work assignments with deadlines to be completed over the three weeks following the workshop (Figure 1), so the learning activity could be implemented in classrooms in the fall 2018 semester. The workload was distributed among the workshop participants and included tasks such as developing student instructions for data manipulation and analysis, finalizing GIS/remote sensing figure overlays, completing biodiversity and NEON PowerPoint teaching slides, writing R code for merging NEON data, creating Excel files with PPPC data, and producing the final student and faculty assessment tools. All draft products were submitted to a document sharing site and were reviewed by all faculty. Once the learning activity products were finalized, they were submitted with an application for IRB review for the project assessment work focusing on student learning (WCU Project Approval #s 1309846‐1 and 1309846‐2). Finally, participants developed a plan for data management and writing of the manuscript.

Given the wide range of faculty expertise and institutional characteristics, it was important to our team that individual instructors be permitted to adjust the learning activity to the needs of their own classrooms, an approach that mirrors how most instructors use teaching modules. In addition to being realistic, this approach has four advantages: (a) It acknowledges that students across classrooms and colleges have different backgrounds and learning needs; (b) it allows individual instructors to adapt the learning activity based on their own backgrounds and their specific course learning outcomes; (c) it allows individual instructors to adjust the learning activity to fit within the time allocated for the activity; and (d) it promotes more in‐depth reflection among instructors about the best ways to adjust the activity to enhance student learning in different settings.

### 2.2 Description of learning activity

The learning activity begins by introducing students to the NEON data portal (https://data.neonscience.org/data‐products/explore) for general exploration and then practice downloading a PPPC field dataset (DP1.10058.001) collected under the Terrestrial Observation System (TOS) for the HARV example site. Students are introduced to the format of NEON field data and the nested plot structure of PPPC sampling design. The exercise includes instructions for either downloading and stacking multiple NEON data files using the “neonUtilities” and “stackByTable()” R code packages or for accessing the data through the NEON API using the “neonUtilities” and “loadByProduct()” R code packages (Figure 2). The exercise also includes an explanation of vegetation variables and biodiversity metrics, and step‐by‐step instructions for manually manipulating Excel spreadsheets to calculate biodiversity indices using Pivot Tables. Workshop participants from the PUI institutions understood the importance of creating versions of the activity with different entry points due to the differing skill sets of our students, software available at our institutions, and the wide range of classes that we teach in any given year. We also wanted to ensure that we created a learning activity that could be adapted for a wide variety of classrooms, ranging from introductory ecology or environmental science courses, to upper‐level GIS, remote sensing, or conservation biology courses. Therefore, in addition to creating instructions that assumed students would be starting by downloading the data from the NEON portal, workshop participants also created Excel files for each of the seven NEON sites, both with and without alpha diversity already calculated. To help instructors in both ecology and spatial science classes link site‐level data with macroscale data, workshop participants also compiled the available spatial data into plot‐level and site‐level shapefiles that could be linked to the Excel files using a common attribute.

### 2.3 Project implementation and assessment data compilation

Of the ten faculty who participated in the workshop, six implemented the learning activity in their classrooms in fall 2018. These faculty used the learning activity in a variety of majors courses at the sophomore to senior (i.e., 200–400) level (i.e., Plant Communities and Ecosystems, Ecosystem Ecology, Conservation Biology, Ecology, Advanced GIS, and Introduction to Remote Sensing). While all of the participating faculty agreed to present certain minimum concepts developed as part of this learning activity (see below; Table 1), the degree of detail in which concepts were covered varied considerably, as did the amount of ancillary material, activities, and software used (Table 2). For example, one of the classes went into greater detail about nested plot designs by including field activities focused on these methods. Other classes implemented additional geospatial analysis activities in ArcGIS and/or used the learning activity within the context of a larger class project.

**TABLE 2:** Summary of information about the six courses that implemented and assessed the classroom learning activity. Time spent on the classroom learning activity includes lecture and laboratory (if applicable). Number of NEON sites is the number of sites for which diversity calculations were completed and analyzed. X indicates a specific program was used by students in the course

^a^Course #	Focus	# of Students	Time spent		Homework included	Laboratory component	# NEON sites	Programs used			Description of learning activity
			# of Hours	# of Classes				Excel	R	ArcMap	
1	Ecology	10	9	3	No	Yes	2		X		Students set up nested plots in a field laboratory, toured the online NEON data portal, then used prepared R code to calculate diversity metrics for two cleaned NEON datasets. Students responded to questions about the R output in a worksheet.
2	Ecology	25	3.5	2	No	Yes	7	X			Species–area curves and diversity were covered in a lecture class on community structure three weeks before implementing the NEON macrosystems lab. In the laboratory, students were introduced to macroscale ecology and NEON, they calculated diversity metrics for their assigned NEON site, made a graph of the relationship between latitude and gamma diversity, and responded to questions in a worksheet.
3	Ecology	21	3.5	4	Yes	No	7	X			All four class sessions started with a minilecture related to the day's content, followed by group work time. Any part of the activity worksheets not finished during class was completed for homework. Day 1: introduction to macroscale ecology, NEON, vegetation dataset. Day 2: calculation of diversity metrics for their assigned site. Day 3: closer look at beta diversity (plot versus site) and gamma diversity by latitude. Day 4: wrap‐up, linking the results to the concepts introduced on Day 1.
4	Ecology	20	5	3	No	Yes	7	X		X	Species–area curves and diversity were covered in a lecture class on community structure six weeks before macrosystems labs. In the first laboratory, students were introduced to macroscale ecology and NEON, they calculated diversity metrics for their assigned NEON site, graphed the relationship between latitude and gamma diversity, and responded to questions in a worksheet. In the second laboratory, students made maps, visually assessed spatial patterns in diversity metrics, and responded to questions in a worksheet
5	Geography	8	4	2	Yes	No	1	X		X	Students calculated an index of vegetation health (NDVI) and then completed the NEON learning activity. They then compared the NDVI data for each site to the diversity metrics calculated using NEON data, and made a graph of the relationship.
6	Geography	25	4	2	No	Yes	1	X		X	Students calculated an index of vegetation health (NDVI) and then completed the NEON learning activity. They were also given remotely sensed net primary productivity (NPP) data and then asked to make several map overlays using NPP, NDVI, and NEON vegetation data.

^a^ Course names: 1—Plant Communities and Ecosystems; 2—Ecosystem Ecology; 3—Conservation Biology; 4—Ecology; 5—Advanced GIS; 6—Introduction to Remote Sensing.

Regardless of the context within which the learning activity was introduced, there was a set of standardized material presented by each faculty member that included an IRB‐required recruitment flyer and subsequent consent form, the student preassessment test and survey, lecture material on macrosystems biology and the NEON project, the classroom learning activity, and the postassessment test and survey. Although the order in which each of these steps was implemented was set, the time period over which they occurred was not. The timing of pre/postassessments relative to the use of the learning activity ranged from a minimum of 7 days to a maximum of 12 days, with a mean of 9 days. To provide a unified structural framework across all classrooms, all faculty used the same “minimum concepts list,” which included concepts related to biodiversity metrics, spatial scaling, and NEON (Table 1). The assessment tools were focused on this list, and therefore, all students took the same assessment.

The student pre‐ and postassessment tools (hereafter referred to as pretest and post‐test) were identical. They were created in Google Forms and administered in class online. The tests comprised 10 multiple‐choice questions (Table 3; supplemental material) testing student understanding of concepts related to the activity learning objectives and 13 questions in which students ranked (1–5; very poor, poor, moderate, good, and very good, respectively) their perceived knowledge of various concepts (alpha diversity, beta diversity, gamma diversity, macrosystems, macroscale, scaling up/down, species–area curves, nested plots, and NEON as an organization) and their perceived skills in Excel, R, and ArcGIS. The majority of students in all courses completed and answered all questions in the pretest and post‐test, and students that did not complete both tests were not included in the analyses. In some courses, these assessments were graded, while some were not graded, and others offered “points” for completion regardless of the correctness of their answers. The authors recognize this disparity could introduce bias into the dataset, but believe the students’ answers are relevant. After the semester was completed and course grades had been submitted, nonconsenting responses were removed from the class datasets and personal identifying information was removed from all remaining student pre‐ and post‐test responses in the master dataset provided to the full faculty team.

**TABLE 3:** Topics, concepts, and the level of Bloom's Taxonomy for pre/post‐test assessment questions for the classroom learning activity

Question #	Topic	Concept(s)	Bloom's level
1	Biodiversity metrics	Alpha, beta, and gamma diversity	Remember
2	Biodiversity metrics	Alpha and gamma diversity	Understand
3	Biodiversity metrics	Alpha, beta, and gamma diversity	Apply
4	Spatial scale	Value of analyzing multiple spatial scales	Understand
5	Spatial scale	Scaling up and scaling down	Understand
6	Spatial scale	Scaling up	Understand
7	Spatial scale	Nested plot sampling	Understand
8	Spatial scale	Macroscale	Apply
9	Spatial scale	Species–area curve	Apply
10	NEON	Application to macroscale science	Apply

Lastly, participating faculty completed the faculty project assessment survey and consent form. The purpose of this survey was to collect information about which EREN‐NEON learning activities each faculty member used in their courses; how much time was spent on lecture, laboratory, and homework; what software programs were used; and information about the course itself (e.g., name, level, and prerequisites).

### 2.4 Statistical analyses

We analyzed differences in overall student performance on the pretest and post‐test using a Wilcoxon signed‐rank test with continuity corrections. We compared student performance on the pretest and post‐test for individual questions using McNemar's tests with continuity corrections using the *gmodels* package in R (Warnes et al., 2015). We analyzed differences in students’ self‐reported understanding of concepts and data skills (using Excel, R, and ArcGIS) on the pretest and post‐test using Wilcoxon signed‐rank tests with continuity corrections. Two courses did not cover the NEON nested plot sampling design (either in lecture or lab), so students in these courses were not included in the analysis for understanding of the nested plot concept. All other concepts were covered in all courses. Five courses used Excel, one course used R, and three courses used ArcGIS (Table 2); students were included in skills analyses only for the programs they used. Wilcoxon's signed‐rank tests were conducted in R version 3.4.1 (R Core Team 2017). For all Wilcoxon's signed‐rank tests, we determined the standardized test statistic (z) using IBM SPSS Statistics version 24.0 (IBM Corp 2016) and calculated Pearson's correlation coefficient (*r*) as a measure of effect size following Field (2009).

## 3 RESULTS

We present results from the analysis of our student assessment data as a “proof of concept” that the learning activity we developed was effective. Due to the diversity of our classroom settings, we focused our assessment on broad concepts. Overall, we found gains in student understanding of macroscale ecology and biodiversity concepts, NEON’s datasets, and skills in data management and use of software programs (spreadsheets, GIS, statistical software), thus meeting the goals of our collaborative effort.

### 3.1 Evaluation of student learning

**FIGURE 3:**
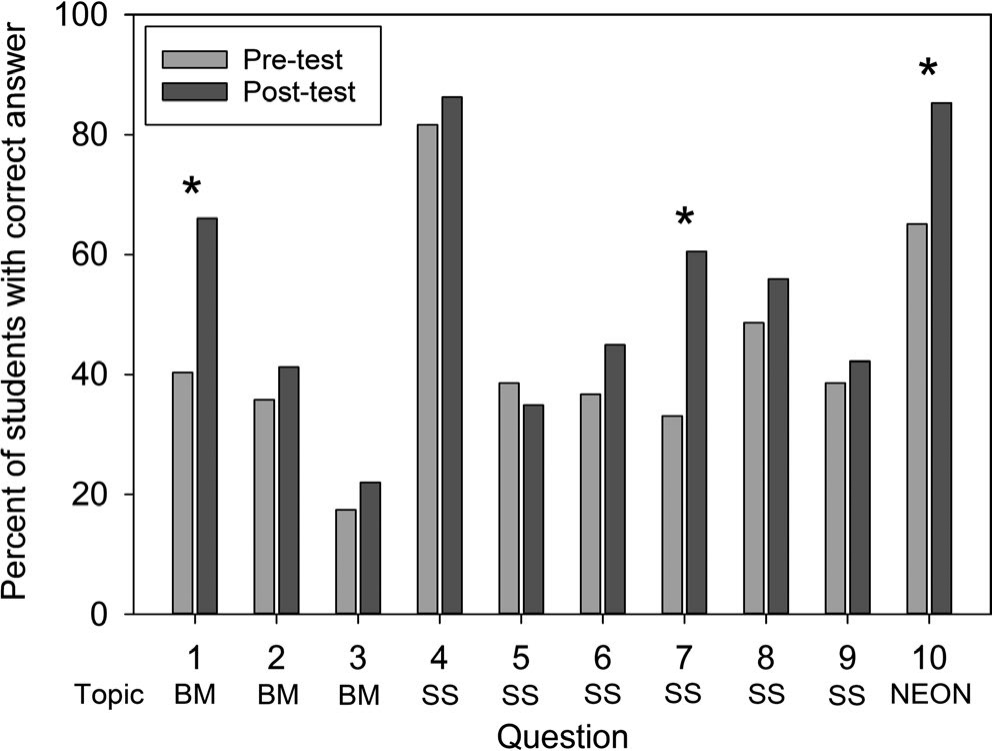
Percent of students that answered each question correctly on the pretest and post‐test. For question topics, BM = biodiversity metrics, SS = spatial scale, and NEON = National Ecological Observatory Network. Q1: *χ* ^2^ = 16.57, *p* < .001; Q2: *χ* ^2^ = 0.74, *p* = .391; Q3: χ^2^ = 0.55, *p* = .458; Q4: χ^2^ = 0.76, *p* = .383; Q5: χ^2^ = 0.32, *p* = .571; Q6: χ^2^ = 1.56, *p* = .212; Q7: χ^2^ = 17.52, *p* < .001; Q8: χ^2^ = 1.02, *p* = .312; Q9: χ^2^ = 0.23, *p* = .635; Q10: χ^2^ = 13.78, *p* < .001. Degrees of freedom = 1 for all questions. *Indicates a significant difference at *α* = 0.05

Students’ scores were significantly higher on the post‐test (mean = 53.94%, Mdn = 50%, IQR = 30) than on the pretest (mean = 43.58%, Mdn = 40%, IQR = 20) across all courses combined (*N* = 109; 85 nonzero differences: V_+_ = 3,064.5, *p* <.001, *r* = 0.37). Student performance was significantly better on the post‐test than the pretest on one question about biodiversity (Q1; *χ* ^2^ = 16.57, *df* = 1, *p* < .001), one question about scaling (Q7; *χ* ^2^ = 17.52, *df* = 1, *p* < .001), and the question about NEON (Q10; *χ* ^2^ = 13.78, *df* = 1, *p* < .001). Student performance did not significantly differ between the pretest and post‐test for the other seven questions (Figure 3).

Students’ self‐reported understanding of all concepts increased significantly after completing the learning activity (Table 4). For most concepts, students reported a “poor” (level 2) median understanding of concepts prior to the learning activity and a “moderate” (level 3) median understanding after completing the learning activity. However, students reported a median “moderate” understanding of species–area curves both before and after the learning activity. Students reported the largest increase in understanding of nested plots and NEON (Table 4).

**TABLE 4:** Summary of students’ self‐reported understanding of concepts and skills. The columns are the concepts and skills covered in class activities, the number of students (with paired pre‐ and post‐tests) that were exposed to a concept or used a program (not the number of students that answered the questions), the mean, median (Mdn), and interquartile range (IQR) of student self‐reported understanding of concepts or skills on the pretest and the post‐test and the difference between the post‐test and pretest, the number of students that had a nonzero difference between the pretest and post‐test rankings, and the results of Wilcoxon's signed‐rank tests with continuity corrections. Students with no difference between the pretest and post‐test rankings are included in median, mean, and standard error calculations. For the Wilcoxon signed‐rank tests, V is the sum of the positive ranks and *r* is the Pearson's correlation coefficient, a measure of effect size

		# students exposed	Pretest			Post‐test			Post‐test–Pretest			# nonzero diff.	V	*p*	*r*
			Mean	Mdn	IQR	Mean	Mdn	IQR	Mean	Mdn	IQR				
Concepts	Alpha diversity	109	2.07	2	2.00	3.41	3	1.00	1.34	1	1.00	88	3,779.5	<.001	0.52
	Beta diversity	109	2.05	2	2.00	3.31	3	1.00	1.27	1	1.00	91	4,013.5	<.001	0.53
	Gamma diversity	109	2.05	2	2.00	3.37	3	1.00	1.32	1	1.00	90	3,951.5	<.001	0.53
	Macrosystems	109	2.33	2	1.00	3.26	3	1.00	0.93	1	1.00	86	3,531.0	<.001	0.51
	Macroscale	109	2.33	2	1.00	3.28	3	1.00	0.95	1	2.00	83	3,324.0	<.001	0.50
	Scaling up/down	109	2.42	2	1.00	3.34	3	1.00	0.92	1	2.00	80	2,983.0	<.001	0.46
	Nested Plots	76	2.14	2	2.00	3.61	4	1.00	1.46	1	1.00	62	1900.0	<.001	0.53
	Species–area curve	109	2.67	3	1.00	3.43	3	1.00	0.76	1	1.00	67	2011.5	<.001	0.38
	NEON	109	1.71	1	1.00	3.23	3	1.00	1.52	2	1.00	96	4,524.5	<.001	0.56
Skills	Excel	99	3.74	4	1.00	3.88	4	1.00	0.14	0	1.00	41	566.5	.051	0.14
	R	10	2.00	2	0.75	3.00	3	0	1.00	1	1.75	8	33.5	.031	0.50
	ArcGIS	53	2.72	3	3.00	3.58	4	1.00	0.87	1	2.00	31	472.0	<.001	0.43

For students that used R and ArcGIS, their self‐reported skills using these programs increased significantly after completing the learning activity (Table 4). Only 41% of responding students reported a change in their perceived knowledge of Excel (41 nonzero differences). Although there was no change in the median perceived knowledge of Excel between the pretest and post‐test, there was a borderline significant increase in individual students’ self‐reported ability to use Excel (Table 4).

### 3.2 Evaluation of approaches to teaching the activity

After piloting the learning activity in a wide variety of courses, the participating faculty met to reflect on what went well across our classes and what we would change in the future. We compiled our notes from this discussion into broad suggested strategies for implementation of our learning activity.

- Given the conceptual challenges presented by biodiversity and scaling concepts, the highest gains in learning are likely to occur when students are given sufficient class time to fully develop their understanding.
- Delivering the material over more than one class period helped students better digest the material (as opposed to being overloaded with new material all at once).
- In classes that had a field component, introducing the concepts of nested plots and measuring vegetation in nested plots in the field before the learning activity helped enhance the ability of students to understand how the NEON data were gathered as well as biodiversity and scaling concepts.
- The timing of the pre‐ and post‐tests could be important to student performance (e.g., Anderson et al., 2020). For example, it is likely better to avoid giving the postassessment tool immediately after spending several hours in class on the learning activity when students are drained.
- If administering the pre‐ and post‐tests online, it may improve student performance to encourage them to use scrap paper so they can write notes or perform calculations to flesh out their answers. Completing the higher‐level quantitative and conceptual questions on the assessment may be difficult for many students without using scrap paper.
- When administering the skills portion of the assessment tool, we recommend revising it to ask about specific skills to obtain more fine‐grained information about self‐reported student learning. For example, instead of asking “On a scale of 1 to 5, please rate your ability to use Excel,” as we did in our generalized assessment tool, ask “…please rate your ability to calculate a mean in Excel” or “… please rate your ability to use Pivot Tables,” an Excel function that was unknown to most students prior to the learning activity and that many students struggled with during the activity.

While some of these observations may seem obvious, we believe their thoughtful implementation would result in improved learning and assessment.

## 4 DISCUSSION

Our learning activity improved student understanding of biological diversity, biodiversity metrics, and patterns of biodiversity across several spatial scales—concepts that can be challenging for undergraduates (Navarro‐Perez & Tidball, 2012). The learning activity introduced students to macroscale ecology and built student skills in working with large datasets and performing basic quantitative analyses, skills that are essential for the next generation of ecologists (Bauerle et al., 2011; Austin, 2018). Our pre‐ and post‐test results demonstrated statistically significant student knowledge gains in biodiversity and scaling concepts, as well as self‐reported technical skills gains in using R and ArcGIS. Students also gained a strong awareness of NEON’s support of macroscale science.

### 4.1 Developing large dataset learning activities

We have several broad recommendations for others to consider when creating similar learning activities to share widely with the larger teaching community. It is important to make the learning activity easily adaptable to individual classrooms (Gould et al., 2014; O’Reilly et al., 2017). To achieve this goal, we suggest providing guidance on various entry points into and exit points out of the exercise, background information in a form that can be easily modified (e.g., slides with notes) for different types of courses, and recommendations on how to implement the activities. Providing teaching materials with varying entry points not only allows for their adoption across a wide variety of courses, but also improves accessibility for faculty who may have varying degrees of comfort working with large datasets, NEON data, or certain software programs (e.g., R, ArcGIS; Bonner et al., 2017; O’Reilly et al., 2017). For example, in classes that focus on learning R, instructors can use the resources provided by NEON to download and organize the data prior to use, while in classes that may instead focus on learning Excel, instructors can use the instructions for creating and working with Pivot Tables. Likewise, in traditional ecology courses the focus may be on the results of the biodiversity analyses, while in a GIS or remote sensing class, the focus may instead be on the relationships between biodiversity and broader‐scale environmental variables.

Regardless of the focus or entry point, all documents should be in formats that are easily edited, which will make it simpler for instructors to adapt and modify the learning activities to fit their classes. In learning activities that involve lengthy descriptions of steps to take, students may get “lost” trying to follow the steps and forget the point of that part of the learning activity (Gould et al., 2014; O’Reilly et al., 2017). An annotated, but brief, outline and summary of major and minor steps and what each step involves and accomplishes should help this issue. It is important, however, to provide the amount of step‐by‐step details necessary for students to achieve the specific learning outcomes for each project and/or course.

The expertise of both NEON staff scientists and PUI faculty was essential to developing this learning activity. NEON staff scientists provided invaluable guidance on downloading, managing, cleaning, and analyzing NEON data. Faculty experience in teaching difficult ecological and quantitative concepts to undergraduates helped guide the team toward a simpler, more accessible activity, with multiple entry and exit points. This collaboration highlights the importance of funding collaborative projects and workshops such as this, both to help interested faculty feel more comfortable using NEON data and to help NEON scientists understand the ways that their data are being utilized (Gould et al., 2014). After this workshop, many of the PUI faculty participants went on to work with additional NEON datasets in their research and other collaborative teaching projects, demonstrating the compounding nature of these investments in faculty training.

### 4.2 Incorporating large datasets into undergraduate classrooms

Conducting classroom learning activities that use large datasets collected over broad spatial scales may address the challenge of teaching certain complex concepts, but these activities can be difficult for instructors to both develop and implement in the classroom (O’Reilly et al., 2017). Designing, teaching, and implementing data‐intensive activities are time‐consuming, both in preparation and instructional time. Using data collected and archived from real‐world projects, such as NEON data, is often messy and can require significant processing time to clean the data (e.g., finding and correcting missing values, selecting a subset of the data variables). This time can be spent by the faculty member in preparation for the activity, or by the students during class instructional time. Careful decisions are needed to determine how curated data should be before students use it and how to scaffold assignments to reduce student frustration and create a slightly more gradual learning curve (Langen et al. 2014; Kjelvik and Schultheis, 2019). We did not assess the costs/benefits of these various approaches in this project, but more work is needed to find the optimal point where the benefits of working with real data are outweighed by the costs in the form of class time used for data processing, student frustration, and lack of student engagement.

The significant time investment required to produce large dataset learning activities can be exacerbated by faculty unfamiliarity with large datasets. Some faculty may not be comfortable with some of the newer methods or software (e.g., R) that may be required or recommended for authentic data analysis (Farrell & Carey, 2018; Hampton et al., 2017). The faculty participants in this project were enthusiastic about utilizing NEON data in our teaching, but we found that these data, although extremely rich, were not always accessible in a way that facilitated their use and adoption into our undergraduate classrooms (Hernandez et al., 2012; Strasser & Hampton, 2012). Our collaboration with NEON staff and scientists was extremely fruitful, and helped to soften the learning curve for this project, but we would not have been able to develop this activity without their direct help. Our experience highlights both the need for additional training and mentorship opportunities for PUI faculty (Bonner et al., 2017) and the need for open‐access data repositories such as NEON to consider ways to improve accessibility for faculty experiencing technology constraints. For example, not all faculty are comfortable with how to download and run an R or Python code to compile data from the NEON portal, so although it is extremely helpful to have those tools, they may not be enough to provide access to NEON data for many PUI faculty (Auker & Barthelmess, 2020).

Some faculty also experience constraints to using large datasets in their teaching at the institutional level. College campuses have varying abilities to support processing of large datasets, as well as to purchase and support various types of software. Having all students running R code simultaneously can sometimes slow classroom internet performance, leading to additional student frustration. Lack of faculty confidence or experience in the tools being used (Farrell & Carey, 2018) combined with inconsistent technology support at smaller colleges present a formidable barrier to the implementation of these activities, even if they are well‐designed and easy to follow.

## 5 CONCLUSIONS

We successfully used nested plot NEON vegetation data to develop a flexible learning activity to teach macroscale concepts related to biodiversity to undergraduates in a variety of courses. Learning activities that use authentic field data and multiscalar analysis methods can facilitate undergraduate understanding of macroscale ecology and allow students to begin to understand biodiversity at multiple spatial scales, preparing them to solve pressing global‐scale, interdisciplinary environmental problems such as biodiversity loss. However, significant support may be needed for faculty to adopt such learning activities en masse.

## CONFLICT OF INTEREST

The authors declare no conflicts of interest.

## AUTHOR CONTRIBUTIONS

**Diane M. Styers:** Conceptualization (equal); Data curation (equal); Investigation (equal); Methodology (equal); Software (equal); Visualization (equal); Writing‐original draft (equal); Writing‐review & editing (equal). **Jennifer L. Schafer:** Conceptualization (equal); Data curation (equal); Formal analysis (equal); Investigation (equal); Methodology (equal); Software (equal); Visualization (equal); Writing‐original draft (equal); Writing‐review & editing (equal). **Mary Beth Kolozsvary:** Conceptualization (equal); Data curation (equal); Investigation (equal); Methodology (equal); Resources (equal); Writing‐original draft (equal); Writing‐review & editing (equal). **Kristen M. Brubaker:** Conceptualization (equal); Data curation (equal); Investigation (equal); Methodology (equal); Software (equal); Visualization (equal); Writing‐original draft (equal); Writing‐review & editing (equal). **Sara E. Scanga:** Conceptualization (equal); Data curation (equal); Formal analysis (equal); Investigation (equal); Methodology (equal); Software (equal); Writing‐original draft (equal); Writing‐review & editing (equal). **Laurel J. Anderson:** Conceptualization (equal); Data curation (equal); Formal analysis (equal); Investigation (equal); Methodology (equal); Software (equal); Writing‐review & editing (equal). **Jessica J. Mitchell:** Conceptualization (equal); Data curation (equal); Funding acquisition (equal); Investigation (equal); Methodology (equal); Project administration (equal); Resources (equal); Software (equal); Writing‐review & editing (equal). **David T Barnett:** Data curation (equal); Methodology (equal); Software (equal); Visualization (equal).

## Supporting information

Supplementary Material

## Data Availability

All teaching module materials described herein are available on QUBES (Styers et al., 2021, https://doi.org/10.25334/DKBX‐8394). The human subjects assessment data presented in this manuscript cannot be publicly archived, per our Institutional Review Board protocol, but are available upon request.
