# Supplementary material for: Developing a flexible learning activity on biodiversity and spatial scale concepts using open‐access vegetation datasets from the National Ecological Observatory Network
Source: Ecol Evol. 2021 Mar 21;11(9):3660–71. doi: 10.1002/ece3.7385 (PMC8093704; doi:10.1002/ece3.7385)
Supplement: Supplementary file 1 — Supplementary Material [file ECE3-11-3660-s001.docx]

**Supplemental Material**

Pre/post-test assessment questions. Questions 1 through 10 relate to understanding of concepts, and correct answers are in italics. Questions 11 and 12 relate to student perception of their knowledge of concepts and skills.

1. Which of the following definitions is correct?

- Beta diversity measures very large-scale diversity. Example: adding up the number of different insect species in North America.
- Gamma diversity measures local diversity. Example: adding up the number of different types of fish in a lake.
- *Beta diversity measures distinctiveness of the biological communities in a region. Example: comparing the species found in forests at the base of the mountain versus the peak of the mountain.*
- Alpha diversity measures macroscale diversity. Example: adding up the total number of different species on the entire planet Earth.
- None of the above.

2. Which of the following statements is NOT true with regards to measures of diversity like alpha diversity and gamma diversity?

- Diversity measures depend on how well plots are searched to find species.
- *Diversity measures are independent of plot size.*
- Diversity measures change over time.
- Diversity measures are dependent on how many plots are sampled.

3. Which of the following is correct with regards to Table 1?

Table 1. Abundance of tree species (number/plot) in two NEON plots.

| Tree species | NEON Plot1 | NEON Plot2 |
| --- | --- | --- |
| Yellow birch | 10 |  |
| Sugar maple | 15 | 80 |
| American beech | 11 | 10 |
| Hemlock | 8 |  |
| Pin cherry |  | 1 |

- Species richness of Plot1 is 44.
- *Gamma diversity is 5.*
- Beta diversity is 3.5.
- Alpha diversity of Plot2 is 91.
- All of the above are correct.

4. Why is it useful to analyze biodiversity at multiple spatial scales?

- Ecological processes that affect biodiversity – for example, nutrient cycling – operate at multiple spatial scales.
- Measuring biodiversity at multiple spatial scales improves understanding of ecological phenomena such as the spread of invasive species.
- Prioritization of areas for biodiversity conservation may change depending on the spatial scale of analysis.
- *All of the above are correct.*
- None of the above are correct.

5. Which of the following is a correct example of ecologists working at multiple scales?

- Comparing density of dandelions in sixteen 1 m2 plots in each of two different old fields.
- Sampling small mammals in the spring, summer, and fall of 2016.
- Calculating average annual air pollen levels in Wisconsin.
- *Relating average regional air temperature to local stream water temperatures.*
- None of the above.

6. Which of the following examples does NOT show how ecological data collected at the ground level can be “scaled up” to describe ecological patterns?

- Calculating the biodiversity of 1 forest plot and extrapolating it to all forested areas across the northeastern US.
- Estimating plant biomass for a 100 m2 plot from plant abundance in nested 1 m2 subplots.
- Measuring plot level species richness to map species diversity across a region.
- *Measuring salamander diversity and plant biomass in fifteen 100 m2 plots.*

7. Which option describes the appropriate sequence of steps for the nested plot sampling method for generating species-area curves to describe plant community diversity?

- *Establish a 1 x 1 m plot and identify all species within the plot. Establish a larger plot 5 x 5 m that includes the area of the first plot and count all additional species that occur in the larger area. Continue to sample the numbers of species in progressively larger plots (10 x 10 m, 20 x 20 m) that include the area of the earlier plots.*
- Establish a 1 x 1 m plot, a 5 x 5 m plot, a 10 x 10 m plot, and a 20 x 20 m plot side by side. Divide the total number of species in each plot by the plot area and compare the number of species per unit area across the plots.
- Establish a 1 x 1 m plot, a 5 x 5 m plot, a 10 x 10 m plot, and a 20 x 20 m plot, each in a different plant community. Count the number of species in each plot and divide by the area to compare diversity across communities.
- Establish a 1 x 1 m plot and identify all species within the plot. Establish a larger plot 5 x 5 m that includes the area of the first plot and count all additional species that occur in the larger area. Compare these values to those found in a 10 x 10 plot that is within a 20 x 20 plot.

8. Which of these panels (A, B, C, or D) shows the macroscale? Choose the best answer.

Figure: A) Elevation across Salmon Creek watershed; B) Minnesota state wind speeds; C) North American growing-season precipitation; D) Predicted 2100 global temperatures

- A and B only
- A, B, and C only
- *C and D only*
- D only
- A, B, C, and D

9. This is Fig. 3 from a paper from Rutherford and Powrie (2013) entitled "Impacts of heavy grazing on plant species richness: A comparison across rangeland biomes of South Africa." Please ignore the dashed lines and focus on the solid lines, which are from the following study areas: 1) Mopane savanna, 2) Grassland, 3) Nama-Karoo, 4) Thicket, 5) Succulent Karoo, and 6) Kalahari dune savanna. The vertical dashed line represents the maximum sampling area (250 m2) in common with all study areas. What is most likely, given the data depicted in Fig. 3 from Rutherford and Powrie (2013)?

- The species richness is highest in the Thicket (4) study area.
- *Most species in the Succulent Karoo (5) study area will be found if a 250 m2 area within the study area is sampled.*
- A 250 m2 area is sufficient to find almost all species within the Mopane savanna (1) study area.
- The species richness is highest in the Kalahari dune (6) study area.

10. How do the objectives of the National Ecological Observatory Network (NEON) support macroscale science?

- NEON provides plot-to-continental scale observations.
- NEON uses consistent data collection methods at all sites, allowing for comparisons across sites.
- NEON provides field-based and remote sensing datasets for the same areas.
- *All of the above are correct.*
- None of the above are correct.

11. On a scale of 1 to 5, please rate your ability to use the following programs.

|  | 1 (very poor) | 2 (poor) | 3 (moderate) | 4 (good) | 5 (very good) |
| --- | --- | --- | --- | --- | --- |
| Excel | O | O | O | O | O |
| R | O | O | O | O | O |
| QGIS | O | O | O | O | O |
| ArcGIS | O | O | O | O | O |

12. On a scale of 1 to 5, please rate your knowledge of the following.

|  | 1 (very poor) | 2 (poor) | 3 (moderate) | 4 (good) | 5 (very good) |
| --- | --- | --- | --- | --- | --- |
| National Ecological Observatory Network | O | O | O | O | O |
| macrosystems | O | O | O | O | O |
| macroscale | O | O | O | O | O |
| scaling up/down | O | O | O | O | O |
| species-area curves | O | O | O | O | O |
| nested plots | O | O | O | O | O |
| alpha diversity | O | O | O | O | O |
| beta diversity | O | O | O | O | O |
| gamma diversity | O | O | O | O | O |

Example Learning Activity Workflow and Timeline

To help provide a clearer picture of what the students did as part of the learning activity in the classroom, below we describe the workflow and timeline for a 300-level Intro Remote Sensing course (GEOG 324). For this class, the learning activity was implemented as part of a larger class project in which students worked with PPPC field data from the Great Smoky Mountains field site (GRSM). The overarching goal was to examine ecological effects of the wildfires that collectively burned more than 170,000 acres of National Forest and Park lands around western North Carolina, eastern Tennessee, and north Georgia and South Carolina during the autumn of 2016. Students focused on the Chimney Tops 2 fire that burned over 11,000 acres in GRSM, including parts of the NEON field site. Set within a very wet climate and history of infrequent burn intervals, these wildfires provide an unprecedented opportunity to establish baseline measurements for how fire influences forest regeneration, plant growth, and plant community composition. To meet the project goal, students utilized the PPPC NEON data and remote sensing-based indices for assessing fire severity, biodiversity, and ecosystem response (e.g., productivity, or annual NPP) to wildfire in southern Appalachian hardwood forests. The workflow and timeline were as follows:

- October 25, 2018: Instructor distributes NEON-EREN Assessment Project IRB recruitment flyer to students and presents project topic; students begin to think about potential research questions
- October 25 - November 2: Students research data and methods appropriate for project as take-home midterm exam in which students are tasked to think about the general project topic and apply this concept to a research question of their choice
- October 29: Instructor’s colleague solicits IRB consent from students at beginning of lab
- November 1: Students complete NEON-EREN Pre-Assessment; Instructor presents overview of Chimney Tops 2 Wildfire
- November 5-9: Students complete Part I of project (remote sensing activity in which students download Landsat satellite imagery and calculate an index of vegetation health, NDVI, and then explore an annual NPP remote sensing data product provided to them)
- November 12-16: Instructor presents overview of Macrosystems Biology and NEON; Students complete Part II of project (NEON field data learning activity)
  - Students were first asked to explore the NEON data portal
  - Students were then given an Excel file with PPPC data for GRSM in which the data had already been downloaded, combined, and edited
  - Students explored the PPPC dataset and were asked questions about the NEON nested plot design
  - Students were then asked to calculate total number of species in each plot (alpha diversity), the total number of species at each of the seven sites (gamma (γ) diversity), and beta (β) diversity using the PPPC dataset and Pivot Tables in Excel and answer questions about plant biodiversity
- November 19-20: Students complete any unfinished activities and Part III (data visualization activity in which students create various map overlays in ArcGIS using the PPPC, NDVI, and NPP datasets)
- November 26 - December 7: Students complete Part IV of project (small group project based on mid-term exam topics that students are capable of completing in two weeks)
- December 6: Students complete NEON-EREN Post-Assessment and Instructor’s post-project assessment of self, project, and peers
- December 11: Students present posters for small group project during final exam period
- After December 17: Instructor completes NEON-EREN Faculty Assessment and Consent; Instructor to send student assessment responses to colleague to remove non-consenting responses and personal identifying information
- After January 1, 2019: IRB lead removes non-consenting responses and personal identifying information from all surveys except WCU and provide a master copy of all consented pre- and post-assessment responses to JEN Team

While this is but one of the six different ways in which the learning activity was implemented, the instructor was easily able to enter and exit the activity at points appropriate for the specific learning outcomes for this project and its role within this intro remote sensing course.
